# Evaluation and utilisation of privacy enhancing technologies—A data spaces perspective

**DOI:** 10.1016/j.dib.2024.110560

**Published:** 2024-05-31

**Authors:** J.M. Auñón, D. Hurtado-Ramírez, L. Porras-Díaz, B. Irigoyen-Peña, S. Rahmian, Y. Al-Khazraji, J. Soler-Garrido, A. Kotsev

**Affiliations:** a GMV, Department of Artificial Intelligence and Big Data, Isaac Newton 11, Tres Cantos, Madrid 28760, Spain; b European Commission, Joint Research Centre (JRC), Algorithmic Transparency Unit, Inca Garcilaso 3, Seville 41092, Spain; c European Commission, Joint Research Centre (JRC), Digital Economy Unit, Via Enrico Fermi 2749, Ispra 21027, Italy

**Keywords:** Privacy preservation, Federated learning, Homomorphic encryption, Differential privacy, Data spaces

## Abstract

Data sharing has facilitated the digitisation of society. We can access our bank accounts or make an appointment with our doctor anytime and anywhere. To achieve this, we have to share certain information, whether personal, professional, etc. This may seem like a minor cost for an individual user, but actually the data economy as the backbone of a digital transformation that is reshaping all aspects of human life. However, one of the major concerns arises regarding what happens to such individual data; once shared, control over it is often lost. For that reason, users and companies are reluctant to share their data. The European Union, through its European Strategy for Data, is establishing a policy and legal framework for establishing a single market for data in Europe by improving the trust and fairness of the data economy. Data spaces are a commitment to sharing data in a reliable and secure way, but this endeavour should, of course, not be at the expense of privacy rights. In recent years, Privacy-Enhancing Technologies (PETs) have emerged to achieve data sharing and privacy preservation that can address the requirements of data spaces around sensitive citizen and business data. In this work, we review existing PETs and assess their relevance, technological maturity, and applicability in the context of common European data spaces. Finally, we illustrate the benefits of secure data sharing via Federated Learning in a healthcare use case, where the preservation of privacy is a primer requirement and is therefore to be guaranteed.

## 1 Introduction

Data is an increasingly important asset that fuels an ever growing number of applications, ranging from the development of new Artificial Intelligence or Machine Learning analytical models to personalised services and smartphone apps. With the rise of the Internet of Things (IoT), Large Language Models (LLM), and a hyper-connected world, the heterogeneity and volumes of data sources are growing exponentially. However, the fact that there is more data available does not intrinsically mean that this data is readily available for use. There are multiple barriers, often occurring concurrently, that drastically reduce users’ ability to access and utilise existing data. These barriers vary in nature and range from legal issues (e.g., overly restrictive or inappropriate licenses) to technical challenges (such as poor data interoperability, vendor lock-in, complex data exchange standards, and fragmented sharing and data processing infrastructures), semantic inconsistencies (different terms and languages used for data encoding), and inadequate data governance approaches that fail to meet the needs of the various actors in the data economy. The contemporary data sharing landscape becomes even more complex as these challenges often do not occur in isolation. They are closely intertwined, which in turn makes it even harder to utilise the data. Within this context, this manuscript investigates PETs by assess their relevance, technological maturity, and applicability in the context of common European data spaces. Structurally, the article investigates in the remainder of Section 1 the policy and technological context, provides an overview in Section 2 of the state of the art and evaluates the different techniques. This is followed (Section 3) by a health data space use case where we implement a set of necessary steps that allow to use access without disclosing their privacy. Finally, we conclude in Section 4 with a summary of the work done, the challenges and opportunities we identified and an outlook for the future.

### 1.1 Policy context

In light of these cross-cutting issues, multiple significant initiatives have been launched and implemented by different stakeholders at multiple governance levels, ranging from the local all the way to the global, that aim at maximising the social and economic benefits of data by improving the ways in which they are shared, processed and utilised. For instance, on the global level, the United Nations have put forward multiple data-sharing initiatives that aim at establishing partnerships around data in support of the implementation and monitoring of the status of Sustainable Development Goals [82].

Then, on the macro-regional level, in the European Union, an ambitions policy agenda is defined by the European Strategy for Data [23]. Launched in 2020, the Strategy aims at establishing a single market for data in the European Union. This will be achieved through a set of policy measures, including (i) creating of the adequate conditions for interconnecting distributed data infrastructures through common building blocks and agreed standards, and (ii) data governance approaches that are fully in line with the EU societal values and existing legal frameworks. A data space according to the policy provisions [25] *brings together relevant data infrastructures and governance frameworks in order to facilitate data pooling and sharing* with the overall objective to support the establishment of a single market for data in the EU. Several closely linked legal instruments, corresponding to the abovementioned vision, provide the legal “backbone” of the Strategy, including:

• *Data Act* [28] which regulates the access regimes to non-personal data and aims at a fairer access to data by the different actors in the data economy (including Business to Business, Business to Consumer, and under certain conditions Business to Government) data sharing.
• *Data Governance Act* which (i) regulates the work of data intermediation service providers and data altruism organisations, and (ii) has the overall ambition to build trust between the different stakeholders in the data economy - [30].
• *High-value datasets Implementing Act*. Developed under the EU Open Data Directive, it aims at providing access, under an open license and in a machine-readable format, to selected datasets produced by EU public authorities. Those carefully selected datasets are considered to be holding a high socio-economic reusability potential and are therefore referred to as high-value datasets - [26].

Once implemented, the data strategy will provide access to data from multiple sectors, including public, private, citizens and research. Clearly, significant portions of such data cannot be released as open data due to different reasons having to do with data subject rights defined under the General Data Protection Regulation (GDPR) [29], statistical confidentiality, business sensitivity of data holders. Therefore, privacy preservation is an essential requirement for data spaces [3]. In a broader sense, ethical considerations and privacy form a central part of the digital agenda of the EU.

## 2 Privacy Enhancing Technologies - An Overview

Privacy Enhancing Technologies (PETs) are defined by the European Union Agency for Cybersecurity (ENISA) as *a coherent system of information and communications technologies (ICT) measures that protect privacy by eliminating or reducing personal data or by preventing unnecessary and/or undesired processing of personal data; all without losing the functionality of the data system* [27]. In other words, they aim to extract value from data without compromising data privacy.

There is no one-size-fits-all PET technique or tool that fulfils this definition. Therefore, the goal of this section is to explore the potential of PETs in different scenarios that promote data sharing without loss of privacy. During this review, we will explore not only the techniques but also their relevance to data space scenarios, the degree of usability, the real-world use cases enabled, and a list of implementations along with their references. Finally, this information is summarised in Section 2.8 using the well-known Technology Readiness Level (TRL) metric, originally developed by NASA in the 1980s and afterwards tailored in the EU for the needs of the Union's framework research programmes [42].

In this section, we address the landscape of PETs, review their main approach, current status, and the applications with the goal of disclosing their applicability and maturity level using the technology readiness level (TRL) indicator [42]. In this state of the art, we restrict ourselves to the analyses of PET individually, however, there are cases where combinations of PET, for example the combined use of a Federated Learning layer and a superimposed Differential Privacy layer, would provide additional safeguards for the original data, however at the expense of an increased complexity and precision of the output.

### 2.1 Secure multi-party computation

Secure Multi-Party Computation (SMPC) [31] is an umbrella term encompassing techniques that allow mutually distrusting data holders to jointly compute a function *f* without revealing more than the function's output:

(1) $$f\left(x_{i}\right)\left(i=1,2,...,p\right),$$

where *x_i_* are the input of parties (data holders).

It is suited for data-sharing scenarios where the data holders share a common goal. Recent advancements in algorithms, network speed, and computing power have made SMPC suitable for real-world usage [56]. However, SMPC suffers from performance overhead due to communication costs, making it unsuitable for large-scale machine learning models, terabyte-sized datasets, or complex real-time applications. Despite this drawback, SMPC provides strong security guarantees based on demonstrated cryptographic protocols such as secret sharing [76], requiring attackers to compromise a majority of computing nodes to disrupt the computation or obtain private data. Some protocols have a costly pre-processing phase, which improves the performance of the actual computation. A *trusted dealer*, that might in the case of the EU policy context be a data intermediation service provider as defined by the Data Governance Act [30], can make these protocols more efficient, at the risk of adding a single point of failure [7,50].

This PET has been used by a variety of applications, including by the Estonian Information Technology and Telecommunications Association (ITL) to compute the statistics and metrics related to the number of employees and labour costs. For that, the members of the association computed these operations using SMPC [11]. In 2015, the same organisation applied again this technique with the goal of finding any relation between the high dropout rate of college students enrolled in Information and Communication Technology (ICT) and the early hiring in the ICT industry [10]. Other real-world applications include encrypted databases, secure auctions, or key management [5]

SMPC has several implementations, both open-source and proprietary. They range from academic implementations of cryptographic protocols to production-ready applications with a focus on real-world deployment (see e.g. [75,49,53,77]). Nevertheless, these tools are not widely adopted and do not seem to be backed up by a broad community of developers and early adopters.

### 2.2 Federated learning

Federated Learning (FL) is a distributed computing approach that preserves data privacy and confidentiality when multiple organisations collaborate to obtain a shared machine learning model [47,40]. In FL, models are trained on separate servers (nodes) without data leaving their storage locations, ensuring data accessibility only within each organisation. Unlike traditional machine learning, FL moves models to the data instead of moving data to a central location. Each node builds a local model and periodically sends its parameters to a central server, where they are aggregated into a global model. The updated global model is then sent back to the nodes for further iterative learning, ending after a predetermined number of rounds. FedAvg [60] is one of the most famous aggregation strategies, if we consider the optimisation of the loss function in machine learning

(2) $$\min_{w\in R^{n}}f\left(w\right),\text{where}\;\;\;f\left(w\right)=\frac{1}{n}\sum_{i=1}^{n}f_{i}\left(w\right),$$

and *f_i_* (*w*) = Loss(*x_i_,y_i_;w*). The loss of prediction on example (*x_i_,y_i_*) with model parameters *w*, while using FedAvg equations are rewritten in the following form:

(3) $$f\left(w\right)=\sum_{k=1}^{K}F_{k}\left(w\right),\;\;\;\;\;\text{where}\;\;\;F_{k}\left(w\right)=\frac{1}{n_{k}}\sum_{i\in P_{k}}f_{i}\left(w\right)$$

with *K* the number of nodes (*k* = 1*,*2*,…,K*) and *P_k_* the set of indexes of data points in client *k* with *n_k_* = ∥*P_k_*∥.

In FL, having Independent and Identically Distributed (IID) data is desirable, but rarely holds in practice due to variations in data size and distribution across clients [88]. FedAvg has no converge guarantees in this situation. The resulting model might be biased, inaccurate, or generalize poorly [88]. This can be mitigated by more advanced aggregation algorithms such as FedProx [55], which dynamically weights the contribution of each node, or FedOpt [72], which runs optimisation on the server by extracting a gradient from the node updates.

Whereas this technology is recent (in 2016, B. McMahan et al., published the first approach to FL [60]) compared to the other technologies covered, it has been widely adopted due to the thousands of applications of deep learning. One of the domains where FL has had a great adoption is the health sector, since the preservation of the privacy of patients is mandatory [73], but also in more daily applications such as mobile keyboard prediction [39].

From a security point of view, FL protocols may contain vulnerabilities for both, (1) the (potentially malicious) server, which can observe individual model updates over time; and (2) any participant who can observe the global parameters and control what is uploaded. For example, malicious participants can deliberately alter their inputs or introduce stealthy backdoors into the global model [6]. These can be mitigated by specialised aggregation functions which try to detect malicious parties and mute their contributions [59]. By combining FL with other PETs (for example, using SMPC for aggregation) the participants can achieve privacy against a malicious server [12].

Efficient communication methods are essential to minimise information transfer, either by reducing communication rounds or message size. Variability in hardware, network connectivity, and power supply requires faulttolerant FL platforms that anticipate low participation, tolerate hardware differences, and handle device dropouts. Several solid implementations exist (see [9,41]) for running in thousands of clients/servers, another feature that has allowed both research groups and private companies to develop solutions without the need to have low-level knowledge of it.

### 2.3 Differential privacy

Differential Privacy (DP) [20,84] is a PET technique that adds noise to data to prevent individual identification from databases when queried using aggregation functions such as mean, sum, or variance. This noise ensures the preservation of statistical properties in the global dataset, guaranteeing a certain level of privacy for specific queries. This guarantee is based on the idea that ensuring query invariance even when removing a record from the database protects against membership and reconstruction attacks, regardless of the adversary's side knowledge about individuals.

Formally, DP is normally defined as follows: a randomised algorithm *M*, is (*ε,δ*)-differentially private if for all databases *D* and *D*′ they differ by at most one row,

(4) Pr[M(x)∈S]≤exp(ε)Pr[M(y)∈S]+δ,

where *M* is a randomised algorithm, *S* means all potential output of *M* that can be predicted, *x* are the entries in the database *D* and *y* the entries of database *D*′, *ε* the maximum distance between the same query on *D* and *D*′ and *δ* the probability of information accidentally being leaked.

The tuple (*ε,δ*) is also known as the privacy budget because it controls the level of privacy (noise added) but also the level of utility, i.e. *ε* → 0 guarantees strong privacy but the output of the probability distribution may be useless.

A practical concern about DP is how it works in a scenario where many users (or a single user which performs several analysis on the same database) need access to the data, with the associated risk that an attacker may combine the outputs with the goal of revealing sensitive data. This scenario is addressed by the Composition Theorem (see [48] which establishes that the composition of *k* queries, each (*ε,δ*)-differentially private, is bounded to (*kε,kδ*) or in other words, privacy is degraded with the number of analysis, but at the same time, it helps to define the overall budget of the system.

In addition, DP is not limited to statistical queries, it has successfully been applied in Deep Learning and Federated learning to protect privacy by adding noise while ensuring that the model still gains insight into the overall population, providing predictions that are accurate enough to be useful, while makes it tough for the adversary to make any sense from the data queried.

There are several use cases where DP has been applied. In 2020, the U.S. Census Bureau developed a system based on DP where a certain amount of noise was added to prevent Census responses from being linked to specific individuals from the database [51]. Another important example is the one announced by Apple, where the company guarantees the privacy of their users by applying local DP [79]. DP provides protection that is robust to a wide range of potential privacy attacks such as re-identification or record linkage, protecting also the cumulative risk from successive data releases but the practical implementation of DP raises concerns about epsilon selection, privacy budget, and sensitivity estimation.

In terms of usability, the mathematical and computational complexity of DP is lower compared to SMPC and homomorphic encryption (HE), which has led to several implementations in a short time available to the general public [32].

### 2.4 Homomorphic encryption

Homomorphic Encryption (HE) allows performing operations on encrypted data instead of raw data. The encryption is based on public-key cryptography, where a public key encrypts the data and only the private key owner can decrypt it. A scheme is defined as homomorphic encryption if

(5) $$E\left(m_{1}\right)\;▴E\left(m_{2}\right)=E\left(m_{1}\;▴\;m_{2}\right)\;\;\;\;\;\forall m_{1},m_{2}\in M$$

where M is the set of messages and *E* is the encryption algorithm over an operation ▲. This scheme is characterised by four main steps: (1) KeyGen: where a set of (public/private) keys are used to encrypt and decrypt; (2) Enc: the process of transforming the input from plaintext to cyphertext, typically using a public key; (3) Dec: the process of transform the input from cyphertext to plaintext, typically using a private key; (4) Eval: performing the operation over cyphertext, the output must guarantee the preservation of the format, that is, cyphertext.

HE is a powerful technique, but this is not free. The main drawback, and one of the main reasons why it is not widely adopted, is the computation overhead. HE, as with any PET technique, is an extra layer of security and this layer has cost and some limitations. The theory establishes that HE can perform any computation [33] (this is known as Fully Homomorphic Encryption), but there are other HE schemes, such as Somewhat and Partial HE where the number and the type operations (sum, multiplication) are limited. In general, the latter are usually quicker to execute but they are not so flexible. This performance issue is considered the main reason why HE is almost limited to research projects (see [14] and references therein). However, the state of the art is constantly improving, reducing the cost of FHE by improving the expensive bootstrapping process and making better use of the hardware [17]. For now, real world usage is limited to specific operations which can be performed efficiently. For example, Private Set Intersection (PSI) allows to perform an inner join between two databases. Apple PSI uses this secure computation into its password monitoring system. In its own words: *Password Monitoring is a feature that matches passwords stored in the user's Password AutoFill keychain against a continuously updated and curated list of passwords known to have been exposed in leaks from different online organisations. If the feature is turned on, the monitoring protocol continuously matches the user's Password AutoFill keychain passwords against the curated list.*

Public-key cryptography deals with problems that take so much time to solve that there is no practical solution in a reasonable amount of time. The classic example is to decompose a number into prime factors, for example, we know that 2·3 = 6, and the inverse operation is easy. However, if we take two large prime numbers and multiply them, trying to decompose them again is time-consuming. Without going into detail, new cryptography algorithms are based on the ring learning-with-error (RLWE) problem [71], which is the basis of the public-key algorithms to protect even against quantum computers. Security vulnerabilities in HE often stem from improper protocol usage or poor implementation rather than the protocols themselves.

While HE is one of the most powerful privacy-enhancing technologies, it is still not ready to cover an arbitrary number of real-world use cases. There are some implementations [16,38,44,86], but all of them require a low-level understanding of this technology.

### 2.5 Zero knowledge proofs

Zero Knowledge Proofs (ZKPs) [37] are cryptographic methods that allow one party (called the prover) to prove the validity of a statement to another party (called the verifier) without revealing the secrets that make this statement true. For example, a prover may want to inform that he or she knows a secret password without revealing it. If the prover really knows the password, and both parties follow the protocol, the verifier will be convinced (*completeness*); if the prover is lying, and the verifier follows the protocol, the verifier will detect the lie (*soundness*) with high probability; all this is done in such a way that the verifier does not learn the password or any other information that would allow him or her to impersonate the prover (*zero-knowledge*).

ZKPs have several applications such as data auditing [36,85], where external auditors can verify the integrity of a dataset without giving them access to the data it contains and the accuracy of a machine learning model service [57,87], where a user has no way of verifying that the model used is the one they are paying for. Other applications are related to verifiable computing [69], identity verification [13] and privacy preserving blockchain [8].

In general, ZKPs are more complex techniques compared to other cryptographic technologies [4]. There is no single framework that covers all variations of ZKP and their development. Additionally, the implementation of new use cases with ZKP currently requires specialised cryptography knowledge. Moreover, the security of a given ZKP must be analysed by both the prover and the verifier. The provers must ensure that they are not sending any sensitive information to the potentially malicious verifier; the verifier must ensure that the potentially malicious prover is not producing a fake proof. While proving these properties requires a specialised mathematical background, this work has already been done for all commonly used ZKP, removing this burden from the users. Due to the probabilistic nature of ZKP, a malicious prover has a small chance of success. To mitigate this, the ZKP is running multiple times, exponentially reducing this possibility. There is a natural trade-off between performance and security: more iterations reduce the possibility of failure at a higher computing cost. Finally, yet very importantly, there are risks associated with security leaks that might compromise ZKP during the setup stage, e.g. where sensitive information exposed during the parameter generation process is used on a later stage for forging proofs and deceiving other actors. Given the relative immaturity of the technology, however, there is no real evidence to date of this vulnerability being exploited by attackers [80].

ZKPs are still in an early stage, which means that there are no stable implementations for the general public, requiring, in turn, specialised cryptographic knowledge. We cite some implementations (see [2,58]), but it is noteworthy that most of them are outdated and without continuous maintenance. However, there are ongoing efforts to develop official standards.

### 2.6 Anonymization

Data anonymization [34,62,66] is known as the set of models and techniques aimed at protecting personal or private information in a data collection scenario (relational, graph-oriented, etc.). It involves altering, deleting, or encoding identifiers that can directly reveal personal information or establish a connection between the data in a database and specific individuals or entities. However, data anonymisation has two main drawbacks.

1. Risk of re-identification: By cross-referencing the anonymised dataset with other data sources, an attacker might be able to re-identify the original data subjects [21]. This is not a merely theoretical concern; the strength of these attacks has been proven in real-world cases [63].
2. Loss of utility: When removing or altering too many attributes, the dataset's statistical utility diminishes. For example, the data subject's address or date of birth might be useful to the data analyst, but unavailable due to anonymisation.

Data anonymisation is by far the most mature technology explored and the simplest at a theoretical and technical level. It is widely accepted and used in the real world: sharing anonymised datasets is routine. It does require some specialised knowledge, since *anonymisation processes need to be tailored to the nature, scope, context, and purposes of processing as well as the risks of varying likelihood and severity for the rights and freedoms of natural person* [1]. Existing legislation takes anonymisation into account, greatly reducing the legal and bureaucratic barriers when working with anonymised data. To sum up, data anonymisation has a high degree of usability. There is also an ongoing discussion over the sufficiency of data anonymisation techniques to protect data subject's privacy, and there are multiple real-world examples of successful reidentification attacks in healthcare [21]. Proper data anonymisation greatly reduces the risk of re-identification, but the risk remains, especially for high-dimensional datasets.

Unlike the other technologies explored in this manuscript, data anonymisation relies on not having been proven to be insecure, rather than being proven to be secure. Data anonymisation algorithms, and the privacy measures they provide (k-anonymity, l-diversity, t-closeness) are purely heuristic and they lack solid theoretical grounding that ensures the level of protection or utility achieved.

A prominent approach alternative to traditional anonymisation is the generation of synthetic data which generates a new (synthetic) dataset that statistically resembles the original one. By doing so, instead of hiding or replacing identifiable properties, this technique involves the creation of a new dataset that maintains the statistical properties of the original one [19]. Some newer approaches leverage on the opportunities of large-language models for the creation of synthetic data, e.g. by instantiating and monitoring synthetic personas [43,68].

### 2.7 Trusted execution environment

Trusted Execution Environment (TEE) is not a technique by itself. Instead, it is a secure area within a main processor that protects code and data from unauthorised entities to tamper with what is running [74]. A key feature of TEEs is providing attestation to a remote user to ensure the enclave remains unmodified [61].

Thanks to this hardware security level, TEE has been used, for example, in the area of Machine Learning. One use case is the use of TEE for Machine Learning as a Service (MLaaS) where an ML model is hosted in a TEE and external data consumers can use it knowing that the transferred data will only be used for this purpose [64]. Another interesting use case is related to the aforementioned Federated Learning, where the role of the aggregation server or the clients can run under this enclave [15].

However, TEEs face challenges regarding isolation and the security of confidential corporate data and databases, since there are possibilities of information leakage via attacks that undermine TEE security guarantees [65]. Mitigation involves enhancing memory and I/O access security. Another main concern is compatibility with current systems in which there may be the possibility of partly rewriting their applications for use in TEEs. This is an important handicap when it comes to using this technology naturally and in the same way that we use current mainstream hardware.

If we want to use this technology now, there are some available implementations such as: Intel SGX, ARM TrustZone (a security extension to the ARM architecture with modifications) and AMD Secure Processor.

### 2.8 Technology readiness level

Following the overview of existing prominent PET, we are now ready to look into their Technology Readiness Level (TRL). These levels range from 1 to 9, where 1 refers to fundamental science and technology (the world of ideas) and 9 to systems that have been proven in large-scale operational systems (for example, the Global System for Mobile (GSM) communications). A TRL-5 means the validation of the technology in a relevant environment. Table 1 stems from the analysis carried out in the beginning of Section 2.

**Table 1:** Technology readiness lever per privacy enhancing technique.

	Secure				Zero		
		Federated Differential Homomorphic					Trusted
	Multi-Party				Knowledge Anonymisation		Execution
		Learning	Privacy	Encryption			Environment
	Computation				Proofs		
Technology Readiness Level	6 ∼ 7	9	7	5 ∼ 6	2 ∼ 3	9	6 ∼ 7

In summary, as we can see from Table 1, the most mature technologies are FL and anonymisation. FL is currently used in thousands of mobile phones and anonymisation (without going into the level of privacy guaranteed) is widely accepted by organisations. As mentioned above, DP, SMPC, and TEE have been tested in realistic relevant environments. HE has been used in a relevant environment, but it requires deep knowledge of cryptography and has performance issues. ZKP is the least mature technology and we believe that it still has years of development to go before it can move up the scale.

It is also important to mention that we have analysed these techniques in isolation, i.e. applying one or the other depending on the scenario and its suitability. However, a combination of several techniques is also possible. A practical example that is used right now in commercial applications is the combination of Federated Learning with Multi-Party Computation. FL requires the aggregation of different machine learning models trained on local data. This aggregation can be done in a central server, with some security concerns, or an extra PET technique, namely MPC, can be used to aggregate the models securely [12]. Develops a Secure Aggregation protocol on top of Secret Sharing scheme. We have to remark that, as we add privacy layers, more complex is to develop and end to end solution due to performance and accuracy issues.

According to these results, Section 3 shows a data-sharing experiment using FL to illustrate the benefits of data sharing in the healthcare data space where patient privacy is critical.

## 3 A Health Data Space Use Case

The healthcare domain generates vast amounts of patient data daily, comprising medical records, imaging data, and clinical trial results. These data sources hold valuable insights for improving patient outcomes and for secondary reuse, such as advancing medical research. This underpins the establishment of the European Health Data Space [24] aimed at optimising health data use within the EU.

There is a strong scientific context for the development of this data space and the utilisation of patient data in AI applications. In recent years, AI especially deep learning, has emerged in this domain for various applications. For example, Natural Language Processing (NLP) has been applied to parse and standardise complex documents such as Electronic Health Records [52]. Another example, in this case in the field of computer vision, is the use of convolutional neural network (CNN) to read mammograms and detect malignant lesions, including architectural distortions [54]. In these examples, researchers are carrying out a set of experiments, but always with data that is not theirs, but that of the data subjects (i.e. the patients). There is a constant strive by academics to have access to more data to improve their Machine Learning models, but healthcare data is subject to strict regulations such as the Health Insurance Portability and Accountability Act (HIPAA) [18] in the United States, and the General Data Protection Regulation (GDPR) [29] in the European Union. According to those legal acts, data sharing is often unfeasible.

Thanks to its maturity level, as described in Section 2.8, Federated Learning can help to overcome this issue through accessing more data while ensuring the privacy and security of sensitive patient data. It helps to share the insights learned by Machine Learning models (via the weights of a ML model) without sharing the raw data, but this only solves one of the many problems researches face when we want to share healthcare data: • Data Heterogeneity. Healthcare data can be highly heterogeneous, with differences in data collection protocols, formats, and standards across different healthcare providers. This heterogeneity can make it challenging to collaborate between researchers effectively.

• Quality of Data. The quality of healthcare data can vary widely, and poor data quality can have a negative impact on the performance of the studies.
• Vocabulary. The vocabulary used to express the same concept may vary depending on where the study is conducted, so providers need to agree on which vocabulary to use before sharing data. Initiatives such as the Observational Medical Outcomes Partnership (OMOP) Common Data Model (CMD) ensures that the data is standardised in a way that makes it easy to compare and combine with other datasets.

From a technical point of view, within the context of a health data space, the following issues have to be taken into account:

• Data imbalances. There can be a significant imbalance in the distribution of data across different healthcare providers, with some having much more data than others (non-identical data distribution (nonIDD)).
• Data Privacy and Security. When it comes to individual data, perhaps the most significant challenge is ensuring the privacy and security of sensitive patient data. Although FL does not require the sharing of raw data, the model updates that are shared during the learning process can potentially leak sensitive information leading to potential exploitation by external attackers. The combination of several PETs can help to overcome this issue.
• Infrastructure and computational challenges. FL requires significant computational resources and robust infrastructures that may not be available to healthcare providers, especially to smaller ones. • Capacity. Small healthcare providers would often not have the necessary capacity in house which is needed for the deployment and operation of FL nodes, which in turn requires this work to be outsourced.

Next sections show the value of sharing the knowledge via Machine Learning models with a specific use case and real data distributed across several institutions, demonstrating that is feasible to work in a cooperative environment, keeping the privacy of the patients.

### 3.1 Experiment

In this study, we have designed a simple FL experiment utilising publicly available datasets of skin lesion images, sourced from four different institutions, described in Section 3.2. The experiment focuses on the detection of skin lesions having an image as input (image classification), so it falls into the area of Artificial Intelligence known as Computer Vision. The selected datasets naturally present some level of heterogeneity, reflecting the diversity of data across different institutions. Our primary objective in the experiment is to address the practical aspects of FL in the context of skin lesion classification.

The performance of the machine learning models trained with local data (trained exclusively on data from an individual institution) versus the federated approach will be compared. The goal is *not* to show the best model (in the bibliography the reader can find several references with this objective) with the best accuracy, but to show the challenges in FL and to demonstrate what happens when the institutions collaborate while preserving privacy, thus being in line with the requirements for common European data spaces. With these results we aim to provide a deeper understanding of the advantages and challenges associated with FL and highlight the potential of PET for fostering secure and collaborative data sharing in data spaces, while handling the inherent heterogeneity of data across different institutions.

### 3.2 Materials and methods

#### 3.2.1 Datasets for skin lesion

To carry out the experiment, we have utilised four datasets of skin lesions, which are openly accessible from three distinct sources: HAM10000 dataset [81], PAD-UFES-20 [67] and MED-NODE [35]. All three sources consist of images along with their corresponding diagnosis, originating from four different countries. HAM10000 is collected from two different locations: the Department of Dermatology at the Medical University of Vienna, Austria, and the skin cancer practice of Cliff Rosendahl in Queensland, Australia; PAD-UFES-20 is collected at the Federal University of Esp´ırito Santo (UFES-Brazil); and MED-NODE is collected from the University Medical Centre Groningen (UMCG) in Netherlands. From here on for simplicity, we will not refer to these datasets by the original name, but by the country where it was collected.

As the raw data utilised in this study originate from various organisations, the dataset exhibits diversity in several aspects, including image quality, imaging devices, ethnicity, location, number of patients, and number of images.

#### 3.2.2 Preprocessing

Preprocessing was accomplished in three steps. The first step is to split the HAM10000 dataset into two subsets: Australia and Austria. In this way four different countries: Australia, Austria, Brazil and Netherlands, with eight different lesions: Actinic Kerastosis (akiec), Basal cell carcinoma (bcc), Benign keratosis (bkl), Dermatofibroma (df), Melanoma (mel), Nevus (nev), Vascular (vasc) and Squamous cell carcinom (scc) were separated.

**Fig. 1:**
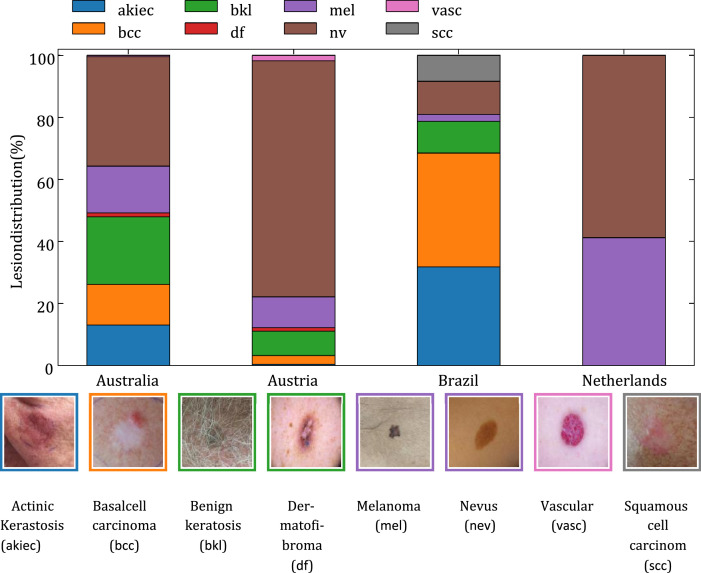
The distribution of skin lesions across four countries with an illustrative sample set.

Fig 1 shows the distribution of skin lesions across the countries. We see that Nevus is the most frequent lesion for Australia, Austria and Netherlands. In other hand, Dermatofibroma, and Vascular and are almost negligible. Squamous cell carcinom only appears in Brazil dataset and Netherlands only has two (Nevus and Melanoma) of eight lesions. The figure demonstrates the usual scenario of non-idd data found in Federated Learning. In this figure we attach one image for each lesion for illustrative purposes.

Table 2 supports the distribution plot showing the number of samples. We see that the Netherlands has only a total of 170 images, whereas Austria is the dataset with the largest volumetry. According to the samples of the images, we decided not to include in the analysis Dermatofibroma (only 30 + 85 images for Australia and Austria respectively), Vascular (only 3 + 139 images for Australia and Austria respectively) and Squamous cell carcinom (only 192 images for Brazil). Therefore, we keep five lesion types through this study: bkl, bcc, akiec mel and nv.

**Table 2:** Number of samples per lesion and per country.

	Australia	Austria	Brazil	Netherlands
Nevus (nv)	803	5902	244	100
Melanoma (mel)	342	771	52	70
Actinic Kerastosis (akiec)	295	32	730	0
Basal cell carcinoma (bcc)	296	218	845	0
Benign keratosi (bkl)	490	609	235	0
Dermatofibroma (df)	30	85	0	0
Vascular (vasc)	3	139	0	0
Squamous cell carcinom (scc)	0	0	192	0

In the second step, the images are normalised to a resolution of 224 × 224 pixels with three channels corresponding to Red, Green and Blud (RGB) and a pixel level in the [0–255] range; and by subtracting the mean (*µ*) and dividing by the standard deviation (*σ*) of each channel.

(6) $$Normalization=\frac{x-\mu }{\sigma },$$

where *x* represents the original image.

Finally, each dataset is split in a training (75 %) and test (25 %) sets where {training} ∩ {test} = 0. A global test dataset is composed from each local tests set. In this way, all lesions are evaluated, and we see how the federated learning model generalises much better than the local model.

### 3.2.3 Neural network and training setup

Convolutional Neural Networks (CNN) have been widely used for classification purposes of images ranging from environmental use cases [78] to healthcare [22], demonstrating that these type of layers within a full neural network architecture give good results in terms of accuracy. However, CNNs often require extensive labelled datasets (while data augmentation techniques are suitable, they are out of the scope of this study). Transfer learning [83] allows models to learn from pre-trained networks, making them capable of learning with smaller datasets and fewer computational resources. Namely, MobileNet v2 [45] has previously been used for classification of skin lesion from HAM10000 dataset [46], so we adopt this architecture in our work.

To address the issue of imbalanced dataset in a multiclass classification problem, a weighted cross entropy loss function *l*(*x,y*) = {*l*_1_*,l*_2_*,…,l_N_*} is used where

(7) $$l_{n}=\sum_{n=1}^{N}\frac{-w_{y_{n}}}{\sum_{n=1}^{N}w_{y_{n}}}\text{log}\frac{\exp\left(x_{n,y_{n}}\right)}{\sum_{c=1}^{C}\exp\left(x_{n,c}\right)}.$$

In this equation *N* spans the minibatch dimension, *x_n,yn_* the input for a specific output *y_n_* and *c* = 1*,*2*,…,C* the classes. *w_yn_* means the weight and in our case, it is the weight of a given class *c* so the minority classes have a weight greater than other classes with more number of samples. Namely, *w_yn_* = {total number of samples}*/*{number of samples of class *c*}, being these values different depending on the specific dataset (see Fig. 1). Notice that the notation used is the one reported by the documentation of PyTorch deep learning framework [70]. Stochastic gradient descent (SGD) algorithm is used for the optimisation of Eq. (7) with a learning rate *η* = 0*.*001. Other parameters of relevance are a batch size *B* = 32 (number of samples utilised before updating the internal model parameters). During the training of the local models with their local datasets a total number of epoch *E* = 50 (number of complete iteration through the whole training dataset) is used. These values are justified since they correspond to the state in which the model no longer learns any more, finding a stabilisation point.

During the training of the federated learning model, the federated averaged strategy (see e.g. (3)) is followed with a total number of rounds *R* = 20 (number of cycles/exchanges of the model between the local institutions and the aggregation server) and *E* = 10 and *B* = 32 per round for every dataset. Notice that this *E* value is lower than its counterpart in the local training (*E* = 50). This is because in our analyses it was observed that if each of the local models had already learned completely, the aggregation of these, and therefore the updating of the model parameters, was worse than if the aggregation was carried out when the model was still in the early learning phase.

#### 3.2.4 Evaluation metrics

The classification of the images is measured using two well-known metrics: F1 score and confusion matrix. F1 is chosen due to the aforementioned imbalanced problem. Namely, f1 reads:

(8) $$F1\;\text{scor}e_{c}=2\frac{\text{Precisio}n_{c}*\text{Recal}l_{c}}{\text{Precisio}n_{c}+\text{Recal}l_{c}}$$

(9) $$Precision_{c}=\frac{TP_{c}}{TP_{c}+FP_{c}}$$

(10) $$Recall_{c}=\frac{TP_{c}}{TP_{c}+FN_{c}}$$

*TP_c_, FP_c_* and *FN_c_* stands for the True Positive (number of instances that were correctly predicted as positive), the False Positive (number of instances that were incorrectly predicted as positive) and False Negative (number of instances that were incorrectly predicted as negative) of a given class *c*. In layman's terms Precision addresses the following statement: of all the positive predictions my model did, how many of them are truly positive? On the other hand, the Recall addresses the next one: of all the actual positive samples, how many of them did my model correctly predict to be positive?

In this study we are working with a multi-class classification problem (an image can be classified in more than two classes), then, a F1 score per class and a weighted-averaged f1 score is used. The weighted score is calculated by taking the mean of all per-class F1 scores while considering each class's support (number of samples of a given class).

To have a visual interpretation of the confusion matrix, it is plotted normalising by class support, in this way the colour bar is uniformly distributed through all the classes (if not, the majority class would take all the colour, implying that the rest of the predictions are incorrect). In the ideal case, the diagonal elements would be dark-blue and the off-diagonal elements white.

#### 3.2.5 Development

The proposed federated system has been implement using Python 3.9 and the open source Flower Federated Learning Framework [9]. Flower is used for the setting up of the federated environment, simulating four different clients and aggregation server. The Deep Learning model is developed using PyTorch 1.12.1.

### 3.3 Results

This section summarises the main results of the experiment, where we compare the defined metrics in two situations:

1. Each hospital trains their model using only its own data, and 2. all the hospitals train a global model using Federated Learning.

**Fig. 2:**
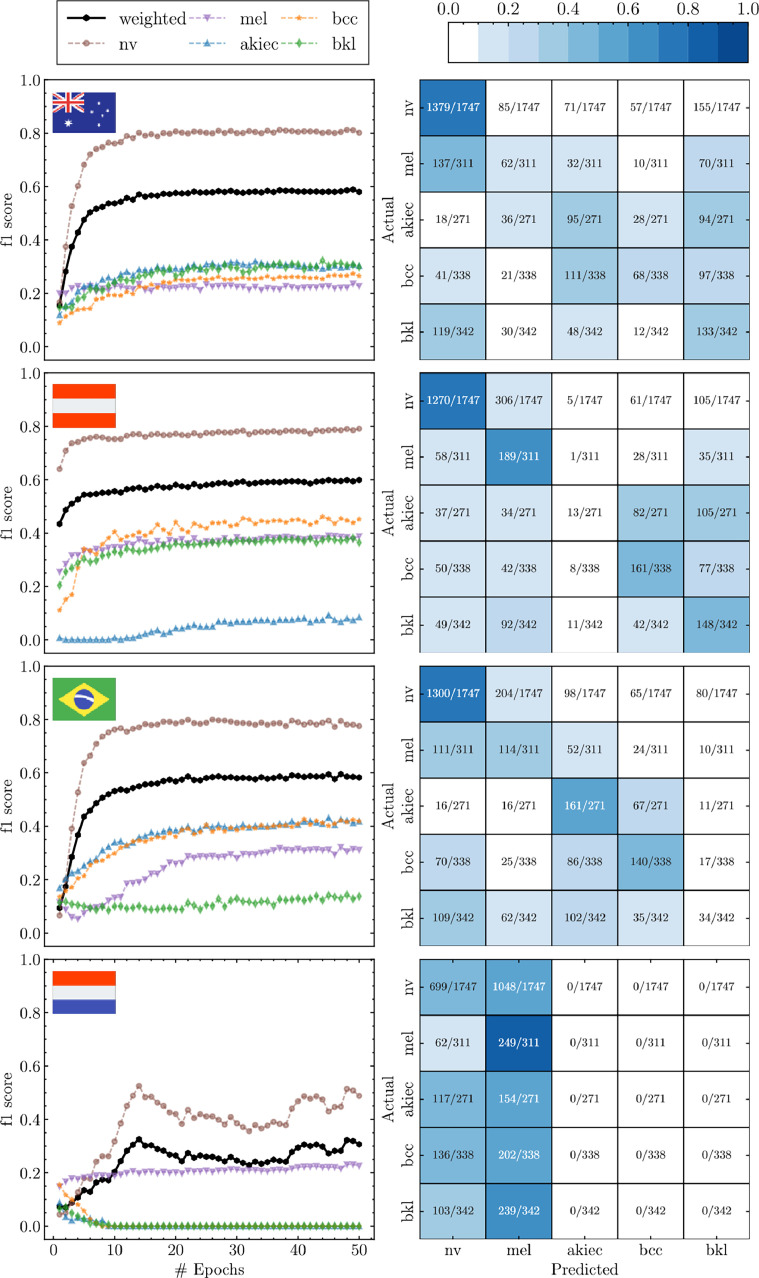
Each row represents the *f*_1_-score (left column) and the confusion matrix (right column at epoch = 50) per country. The *f*_1_-score is calculated per class and their average weighted by the support (number of samples).

Fig. 2 depicts the first situation, showing the *f*_1_-score (left column) and the associated confusion matrix (right column) per country. We can see that, in general, the majority class nv is the best predicted lesion, reaching a maximum Recall_nv_ = 1379*/*1747 ≃ 79 % for Australia. The weighted *f*_1_*_,weighted_* ≃ 0*.*6 for all the countries but Netherlands, having *f*_1_*_,weighted_* ≃ 0*.*3. For the rest of lesions there are different results depending on the country. Austria has issues (white colour in the non-diagonal positions of the confusion matrix) for predicting akiec; Brazil for predicting bkl and Netherlands with akiek, bcc and bkl. These results are not surprising, Table 2 showed that Australia has a relevant number of samples for all lesion but Austria only has 32 for akiec, so the model has not learnt this lesion. Netherlands had not samples of akiec, bcc and bkl in the training set, so the prediction power is null (*f*_1_*_,_*_akiec,bcc,bkl_ → 0). At this moment the conclusion could be that it is not possible to learn lesions without access to the raw data.

**Fig. 3:**
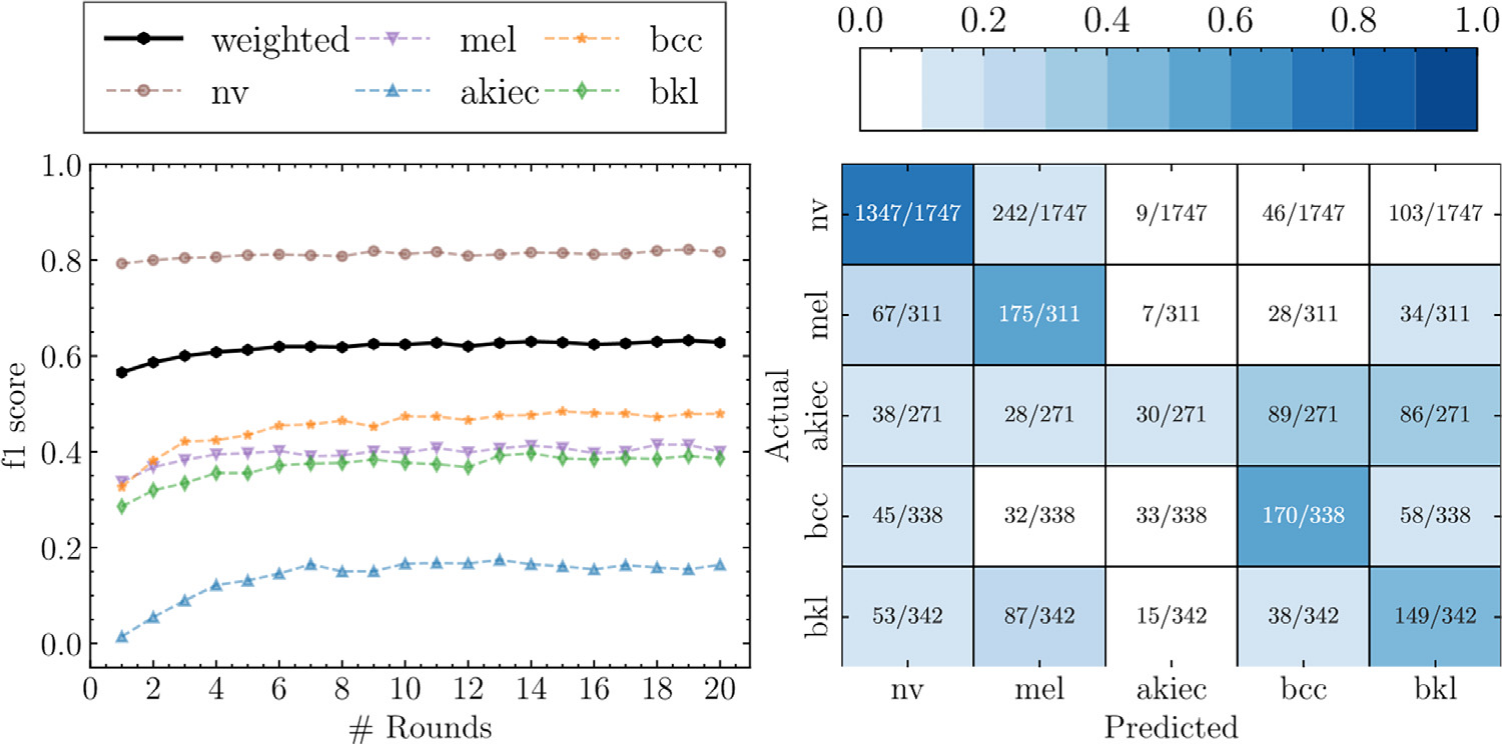
*f*_1_-score (left) and the confusion matrix (right at round = 20) for the Federated Learning setup. The *f*_1_-score is calculated per class and their average weighted by the support (number of samples).

Fig. 3 shows the results for the Federated Learning setup. In this situation only the weights of the model are shared and aggregated in every communication round. In general, we can see that the metrics are of the same order or better than its local training counterpart. For example, now Netherlands has a model capable of predicting lesions that, using only data from itself, would not be possible, that is

(11) $${\text{Recall}}_{akiec,bcc,bkl}^{Netherlands}\ll {\text{Recall}}_{akiec,bcc,bkl}^{FL}.$$

This enhancement of the prediction power is one of the main conclusion of this study. We find similar situations for Brazil, being possible to predict bkl and Austria for akiec. However, it should be noted that for some very specific cases, the model has been degraded. This is the case for Australia and Brazil with akiec, where the Recall_*Australia,Brazil*akiec_ *>* Recall_*FL*akiec_, but for the rest of lesions

(12) $${\text{Recall}}_{nv,mel,bcc,bkl}^{Australia,Brazil}\ll {\text{Recall}}_{nv,mel,bcc,bkl}^{FL}.$$

This approach of sharing the model instead of raw data maintains the integrity of federated learning's fundamental principle: preserving user privacy. It circumvents the need to share sensitive raw data, instead of only sharing non-sensitive aggregated data metrics. Thus, we can harness the potential benefits of broader data exposure while still retaining the fundamental privacy protections that federated learning affords.

## 4 Discussion and Conclusions

This work presents an evaluation of the most prominent PETs within the context of emerging data spaces. The results indicate that these techniques hold significant potential for accessing and utilising sensitive private and personal data. However, the maturity levels and technology readiness of these techniques vary considerably. Additionally, no single technique can be universally applied to all data space use cases. Each potential solution has its own distinct advantages and disadvantages, and its application is associated with certain costs, such as distortion of the end result or computational expenses.

In the healthcare use case described in Section 3, we have demonstrated FL's potential to deliver effective machine learning models while preserving privacy. However, our study also highlights significant challenges in realising this potential, mainly due to the heterogeneous nature of the datasets and class imbalances. Differences between the datasets, arising from their varied sources, were found to hinder the performance of the federated model, indicating a critical need for extensive preprocessing and data homogenisation. In particular, addressing the problem of class imbalance emerges as a critical consideration for improving the model's ability to learn effectively across diverse and unevenly distributed classes. Further experimentation with different model aggregation strategies could potentially lead to better results in the federated environment. Although FL shows promising prospects in the field of privacy-preserving machine learning, these results emphasise the necessity for careful data preparation and strategic handling of class imbalances to fully leverage its capabilities. Our study sets the stage for future research aimed at developing more robust PET capable of addressing those challenges and delivering enhanced performance in diverse data space scenarios. Within that context, the role of data intermediary and data alrtuism organisations, as they are defined by legislation [30] are to be investigated.

Finally, another important aspect to be taken into account when conceptualising applications requiring the use of PET is the possibility to use more than one technique altogether. This would substantially improve privacy but would also not come without its own specificity and associated costs. Clearly, such an approach has to be further investigated in terms of applicability, maturity, possible drawbacks and appropriate implementation strategies.

## Ethics Statement

The authors have read and follow the ethical requirements for publication in Data in Brief and confirming that the current work does not involve human subjects, animal experiments, or any data collected from social media platforms.

## CRediT Author Statement

Project proposal, J.S-G and A.K.; PET analysis, software and experiment implementation, J.M-A, D.H-R, L.P-D, B.I-P and S.R.; project administration and coordination, J.M-A, Y.A and A.K. All authors have read and agreed to the published version of the manuscript.

## Data Availability

Not applicable (Original data) (JRC Science hub).
